# Navigating Ramadan Fasting Following Laparoscopic Sleeve Gastrectomy: Clinical Insights and Patient Experiences

**DOI:** 10.7759/cureus.94435

**Published:** 2025-10-13

**Authors:** Kareem A Farouk, Mohamed Abdelhalim, Ibtehal Elgaabari, Suzanna Hewitt, Ahmed Hamad

**Affiliations:** 1 General Surgery, Tanta University Hospitals, Tanta, EGY; 2 General Surgery, Tanta University, Tanta, EGY; 3 Surgery, County Durham and Darlington NHS Foundation Trust, Durham, GBR; 4 Department of Food Hygiene and Control, Faculty of Veterinary Medicine, Benha University, Benha, EGY

**Keywords:** bariatric surgery, dehydration, dietary recommendation, gastroesophageal reflux disease, laparoscopic sleeve gastrectomy, protein intake, ramadan fasting

## Abstract

Background and objective

Fasting during Ramadan presents unique challenges for post-bariatric surgery patients. The effects of fasting after laparoscopic sleeve gastrectomy (LSG) remain unclear, particularly regarding symptom prevalence and fasting success rates. This study aimed to evaluate the optimal timing for fasting after LSG and provide guidance for safely observing Ramadan.

Methods

We conducted a retrospective observational study at a tertiary care hospital in Egypt, analyzing patient experiences with fasting after LSG. The study involved 100 patients who had undergone LSG at different intervals before Ramadan (three, six, nine, and 12+ months). Patients completed surveys assessing fasting success rates, symptom prevalence (gastroesophageal reflux disease (GERD), vomiting, dumping syndrome), and medical interventions required. The primary outcome was the ability to complete Ramadan fasting without hospitalization or intravenous fluid administration. Secondary outcomes included symptom patterns, nutritional status, and hydration practices.

Results

Fasting completion rates significantly improved with time since surgery, with patients fasting ≥12 months post-LSG exhibiting the highest success rates. GERD prevalence increased over time, particularly in the ≥12 months group, while vomiting and dumping syndromes remained stable. No deficiencies in calcium, vitamin D, or vitamin B were observed, and no cases of dehydration required hospitalization. Older patients experienced more symptoms than younger individuals, and prior fasting experience positively influenced fasting success.

Conclusions

Post-LSG fasting during Ramadan is safest when initiated at least 12 months after surgery. Long-term monitoring, individualized medical guidance, and hydration strategies are essential to ensure safe fasting in post-bariatric patients.

## Introduction

Ramadan fasting, a religious practice observed by Muslims, involves complete abstinence from food, drinking, and other activities from dawn till sunset for one month. This form of time-restricted feeding significantly alters meal schedules and dietary habits, leading to various effects on cardiometabolic health [1]. Laparoscopic sleeve gastrectomy (LSG) is a popular and effective bariatric procedure for treating morbid obesity [2,3], leading to significant weight loss and improvement of obesity-related comorbidities [4]. The procedure involves reducing stomach size, which may affect a patient's ability to consume large meals or drink large quantities of fluids at once. Ramadan fasting can have a significant impact on patients who have undergone bariatric surgery; however, research specifically examining this topic is limited. Generally, patients undergoing bariatric surgery are advised to follow strict dietary guidelines, which may conflict with Ramadan fasting practices [5].

Several factors should be considered in patients undergoing bariatric surgery, such as BMI, related comorbidities, psychological evaluation, diet, lifestyle, and commitment to long-term follow-up. Weight loss outcomes after bariatric surgery can be influenced by various factors, including pre-existing conditions, such as diabetes. Patients with diabetes tend to experience less weight loss than non-diabetic patients after bariatric surgery [6]. Additionally, genetic factors may play a role, as certain mutations have been associated with reduced weight loss after bariatric surgery [7]. Therefore, this study aimed to evaluate the optimal timing for fasting after LSG and provide guidance for patients to safely observe Ramadan fasting.

## Materials and methods

Study design

This retrospective observational study aimed to evaluate the optimal timing for fasting after bariatric surgery and to provide guidance for patients to safely observe Ramadan. This study analyzed the experiences of 100 patients with ages ranging from 21 to 50 years, who underwent LSG at varying durations before Ramadan (three, six, nine, and 12+ months) in Egypt. Fifty-six of them were females and 44 were males. The participants were surveyed regarding their fasting experiences during Ramadan, including completion rates, symptoms encountered, and any medical interventions required.

Eligibility criteria

Inclusion Criteria

Participants were included in the study if they met the following criteria: (1) had undergone LSG at least three months before Ramadan; (2) were willing and able to share their experiences of fasting during Ramadan; and (3) had complete data regarding fasting completion, symptoms experienced, and any medical interventions required.

Exclusion Criteria

Participants were excluded from the study if they met any of the following criteria: (1) had undergone a bariatric procedure other than LSG (e.g., gastric bypass or adjustable gastric banding); (2) had incomplete data regarding fasting experiences, symptom prevalence, and medical interventions; (3) were unable or unwilling to provide informed consent to participate in the study; and (4) had significant comorbidities such as diabetes mellitus and heart disease.

Participants

A total of 100 patients who met the inclusion criteria were included in this study. The participants were divided into four groups based on the duration of surgery: (1) three months post-surgery: patients who underwent LSG within three months before Ramadan; (2) six months post-surgery: patients who underwent LSG within six months before Ramadan; (3) nine months post-surgery: patients who underwent LSG within nine months before Ramadan; and (4) 12+ months post-surgery: patients who underwent LSG 12 months or more before Ramadan.

Demographic data, including age, sex, and previous fasting experience, were collected from all participants. Additionally, the presence of comorbidities, such as gastroesophageal reflux disease (GERD), vitamin deficiencies, and other gastrointestinal symptoms, was recorded.

Data collection

Data were collected through a structured questionnaire administered to participants after Ramadan. The questionnaire captured the following information: (1) fasting completion: whether the participant completed the full month of Ramadan (yes or no) and whether they experienced any complications requiring hospital admission or intravenous fluids; (2) experienced symptoms: participants reported the occurrence of GERD, vomiting, dumping syndrome, dehydration, and other gastrointestinal symptoms during Ramadan; (3) nutritional status: the presence of calcium, vitamin D, and vitamin B deficiencies was assessed using subjective self-reported symptoms and medical records; (4) hydration practices: participants were asked about their fluid intake during non-fasting hours, with a focus on adherence to recommended hydration guidelines (minimum 1.5 liters per day); and (5) physical activity: participants reported whether they maintained physical activity during Ramadan and any adjustments made to exercise intensity or timing.

Outcome measures

The primary outcome measure was the ability to complete Ramadan fasting without requiring hospital admission or intravenous fluid administration. Secondary outcomes included the prevalence of GERD, vomiting, dumping syndrome, and other gastrointestinal symptoms, as well as nutritional status and hydration practices.

Statistical analysis

Data were analyzed using descriptive statistics to summarize patient characteristics, fasting completion rates, and symptom prevalence across the four groups. Frequencies and percentages were calculated for categorical variables, whereas means and standard deviations (SD) were used for continuous variables. Comparisons between groups were performed using the chi-square test for categorical variables and one-way ANOVA for continuous variables, where appropriate.

Ethical considerations

The study adhered to ethical guidelines for research involving human participants. Informed consent was obtained from all participants, and confidentiality of their personal data was ensured throughout the study. The study protocol was reviewed and approved by the Ethical Committee of the Faculty of Medicine, Tanta University (approval no: 36264PR220/4/23).

## Results

**Figure 1:**
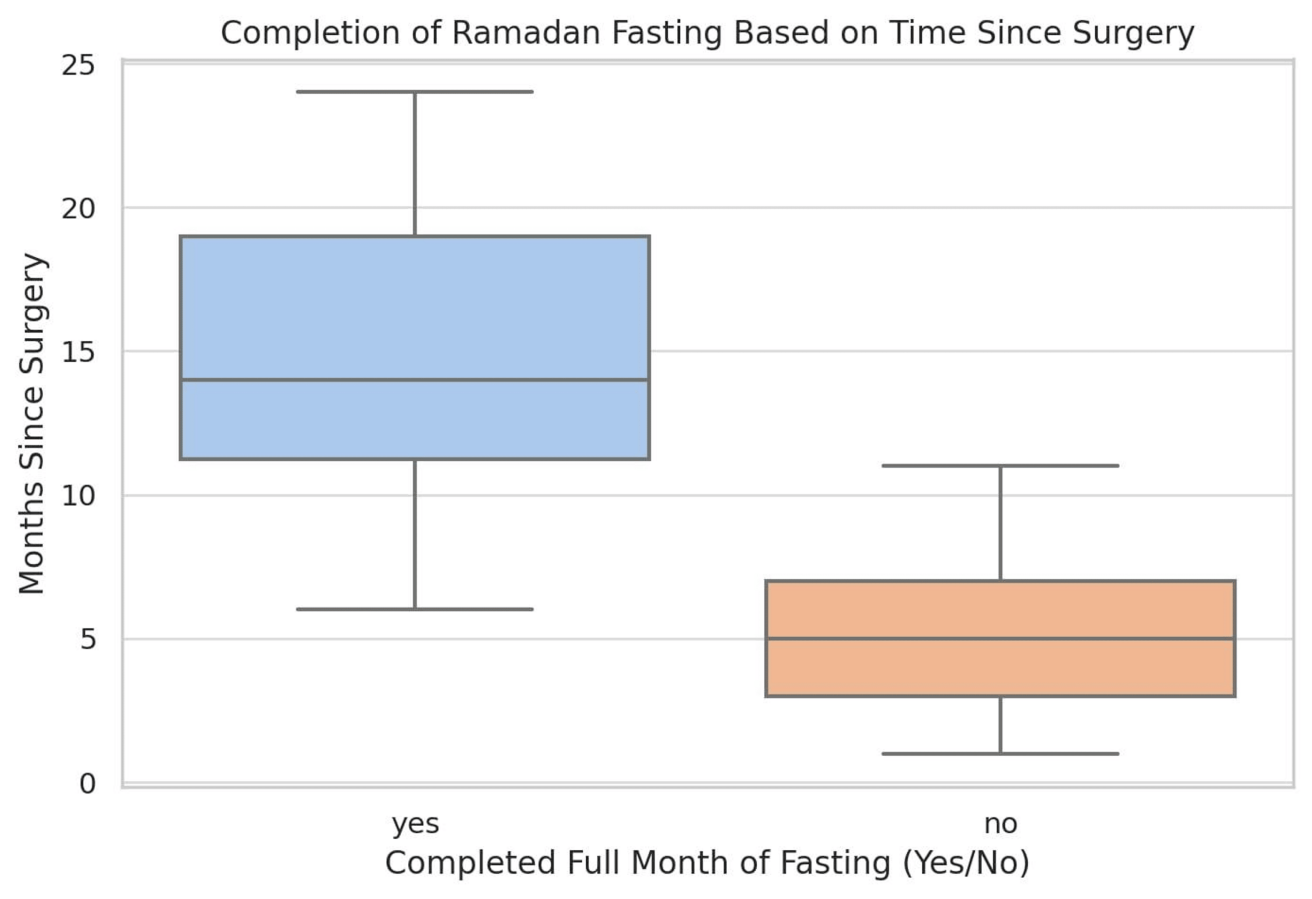
Completion of Ramadan fasting according to the duration since LSG Data are presented as the number (N) and percentage (%) of patients who successfully completed fasting LSG: laparoscopic sleeve gastrectomy

This study analyzed the experiences of 100 patients who underwent LSG for varying durations before Ramadan at three, six, nine, and 12+ months. The findings revealed that fasting completion rates were significantly influenced by the time elapsed since the surgery (Figure 1).

These data suggest a significant association between the time elapsed post-surgery and fasting success. Patients who completed the fast had a longer median duration after surgery, typically ranging between 10 and 20 months, with some extending beyond 24 months. This trend indicates that a longer post-LSG recovery period may enhance patients’ ability to tolerate prolonged fasting. Conversely, individuals who were unable to complete the fasting month exhibited a shorter time since surgery, with most cases occurring within 3-10 months postoperatively. This finding suggests that the early postoperative phases may present physiological challenges that limit fasting capability, due to ongoing metabolic adaptation, altered gastric capacity, or nutritional constraints. The interquartile range for this group also indicated a relatively tight distribution, reinforcing the observation that fasting intolerance is more pronounced among those with less than a year of recovery. Overall, these results emphasize the importance of adequate postoperative recovery prior to prolonged fasting. While individual variability exists, a minimum of 9-12 months post-surgery appears to be a critical threshold for improved fasting tolerance. 

**Figure 2:**
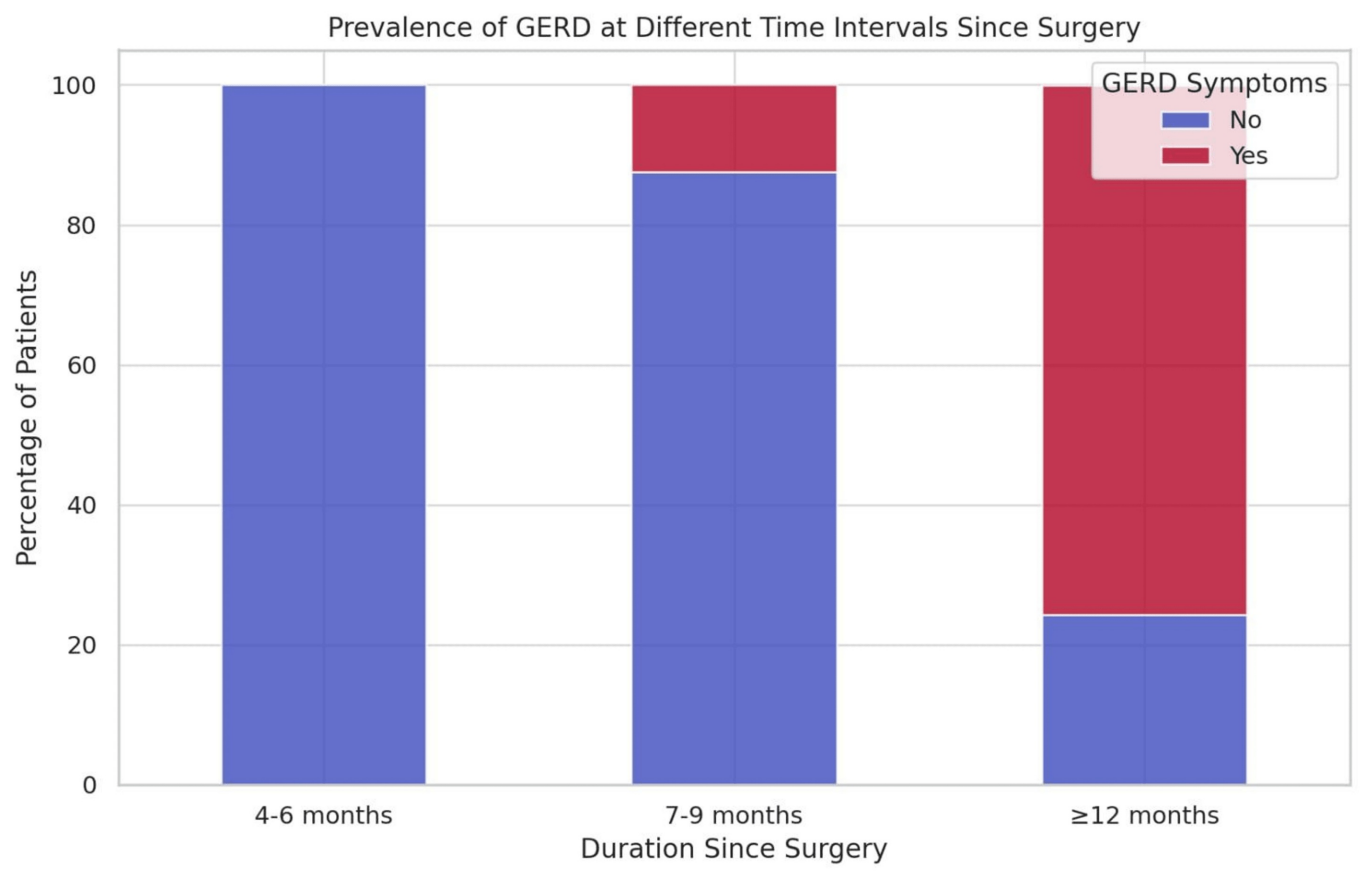
Prevalence of GERD at different durations since LSG Data are expressed as the percentage (%) of patients reporting GERD in each group GERD: gastroesophageal reflux disease; LSG: laparoscopic sleeve gastrectomy

These results suggest that the prevalence of GERD increases with time after surgery. The ≤3-months group had no reported GERD symptoms, indicating that the early post-surgical phases might not present significant reflux complications. However, as time progressed, GERD symptoms appeared more frequently, with a notable increase in the ≥12 months group, where a substantial proportion of patients reported GERD. This trend suggests that long-term follow-up is essential for monitoring GERD symptoms in patients undergoing LSG, particularly beyond the first postoperative year. The increased prevalence at later intervals may be linked to anatomical changes in the gastric sleeve, alterations in esophageal motility, or dietary habits. Further research is required to assess preventive strategies or medical interventions to manage GERD in post-bariatric patients (Figure 2).

These findings suggest a dynamic trend in the prevalence of symptoms across different postoperative intervals. GERD appears to be significantly more prevalent in patients who have undergone surgery for ≥12 months than in those in earlier stages. This could indicate late-onset complications, possibly due to anatomical changes in the stomach, alterations in esophageal motility, or progressive relaxation of the lower esophageal sphincter. In contrast, vomiting and dumping syndrome exhibited a more stable pattern over time, with a relatively lower prevalence across all groups. These symptoms may be more pronounced in the early postoperative period due to dietary adjustments and the body's adaptation to reduced gastric volume. However, as patients adapt their eating behaviors, the frequency of these symptoms appears to remain relatively steady or slightly increase in the long term.

**Figure 3:**
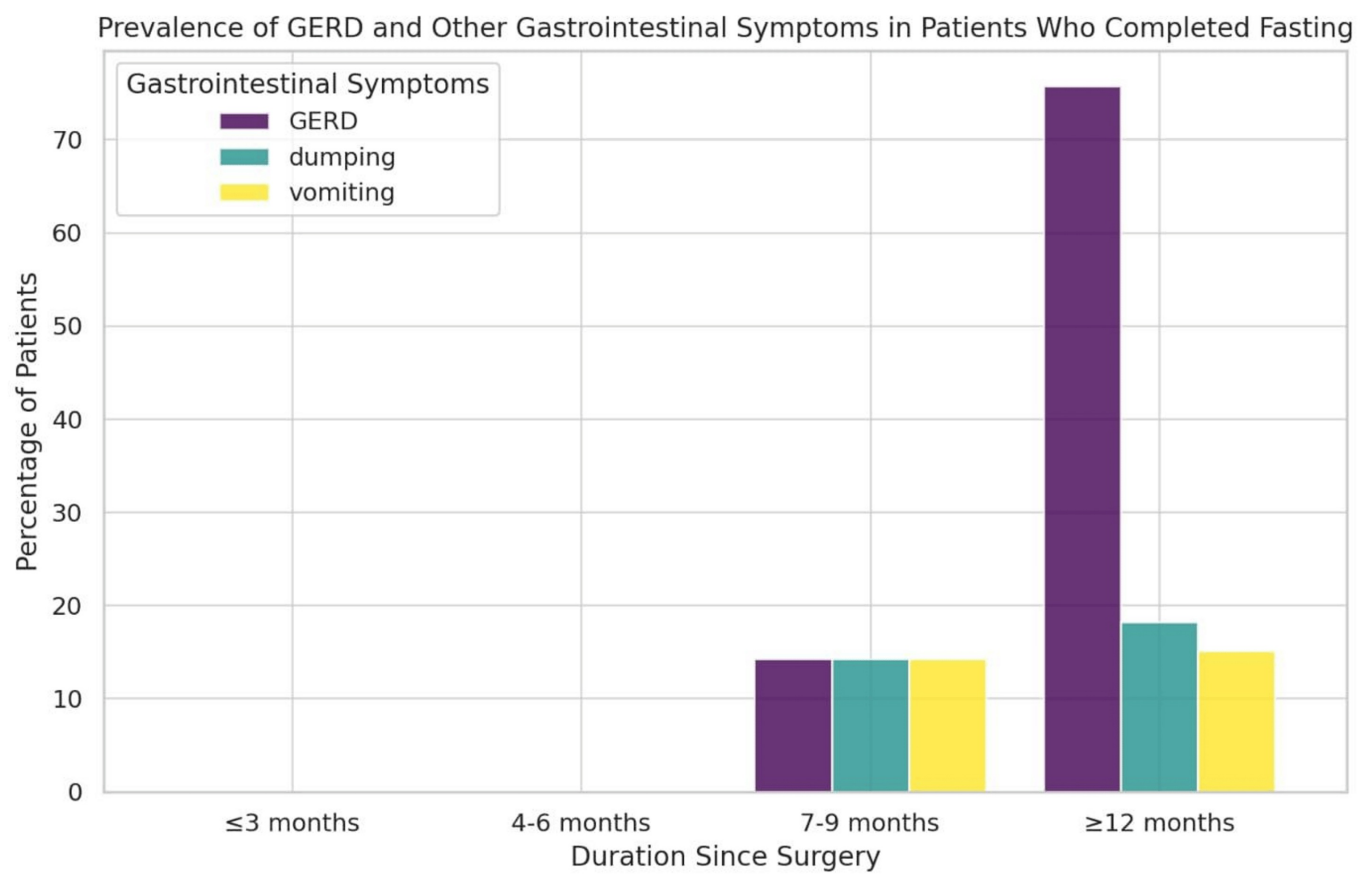
Prevalence of GERD, vomiting, and dumping syndrome at different durations since LSG in patients who completed fasting Data are expressed as percentage (%) of patients GERD: gastroesophageal reflux disease; LSG: laparoscopic sleeve gastrectomy

This study highlights the importance of long-term follow-up in patients undergoing bariatric surgery to assess the persistence or emergence of gastrointestinal symptoms. The substantial increase in the GERD prevalence in the ≥12-month group underscores the need for monitoring and potential medical or dietary interventions to mitigate discomfort and prevent complications. These findings contribute to a better understanding of postoperative symptom progression and reinforce the necessity of patient education regarding dietary modifications and symptom management throughout different recovery stages (Figure 3).

**Figure 4:**
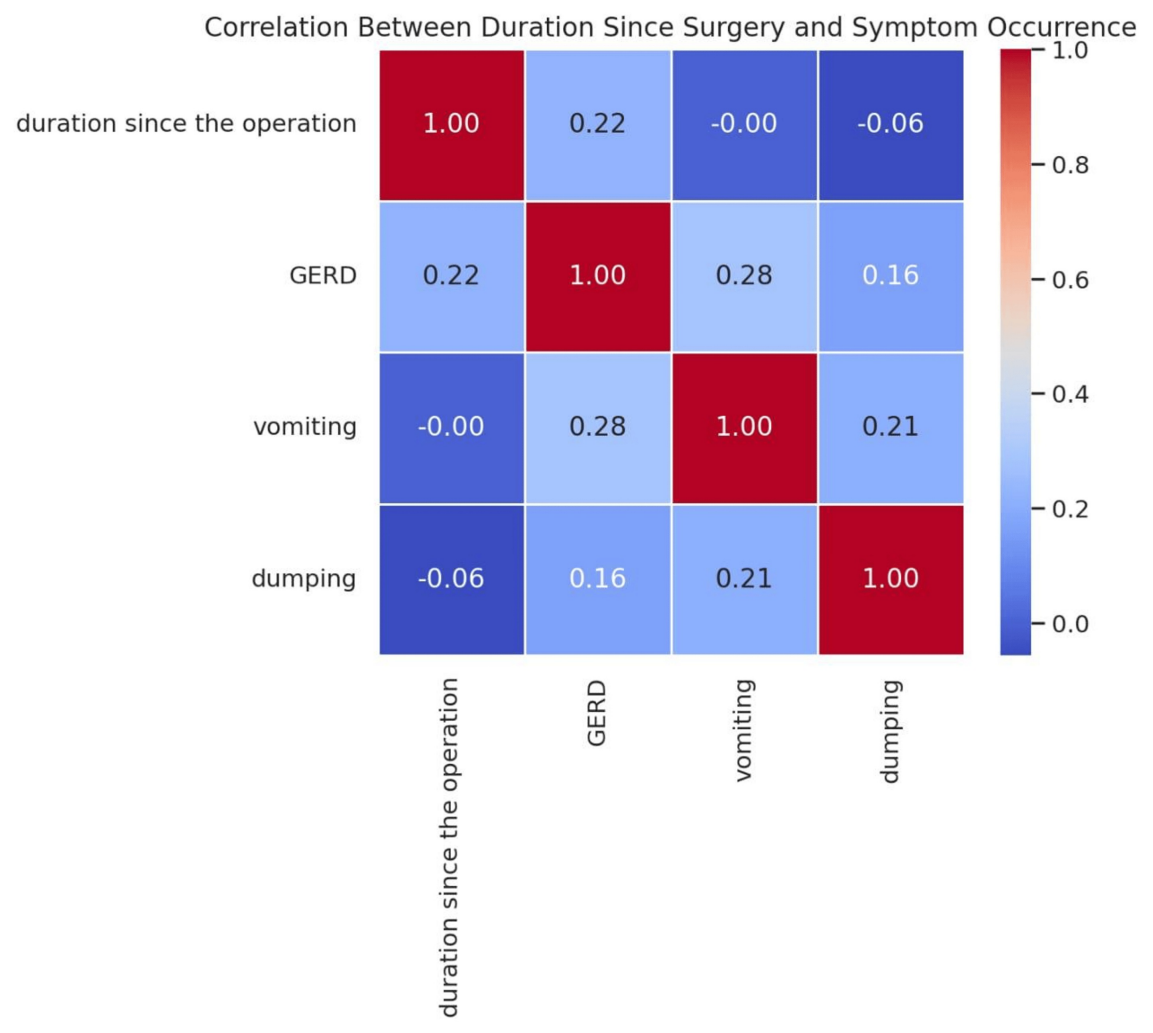
Correlation between the duration since LSG and the occurrence of postoperative symptoms (GERD, vomiting, dumping syndrome) Data are presented as correlation coefficients (r). Statistical analysis was performed using correlation testing (r=1) GERD: gastroesophageal reflux disease; LSG: laparoscopic sleeve gastrectomy

There was a correlation between the duration since surgery and the occurrence rate of three postoperative symptoms: GERD, vomiting, and dumping syndrome (Figure 4).

Based on the results, GERD appears to have the strongest positive correlation with the duration since surgery, indicating that patients who have undergone surgery for a longer period of time are more likely to experience GERD symptoms. Vomiting and dumping syndrome show weaker or negligible correlations, suggesting that these symptoms might be influenced more by other factors, such as dietary habits, rather than the time elapsed since surgery. These findings highlight the need for long-term monitoring of GERD symptoms in post-bariatric patients, particularly beyond the first postoperative year. Future studies should investigate additional contributing factors, such as dietary patterns, gastric motility changes, or weight fluctuations.

Despite concerns regarding vitamin and mineral deficiencies during prolonged fasting, no instances of calcium, vitamin D, or vitamin B deficiencies were observed in the study population. Furthermore, dehydration, a common concern during extended fasting, did not lead to hospitalization in any of the cases. This outcome likely reflects adequate hydration practices among participants, who consumed at least 1.5 liters of fluids daily during non-fasting hours. These findings suggest that when patients adhere to proper nutritional guidelines, they can maintain adequate micronutrient levels, even during fasting.

**Figure 5:**
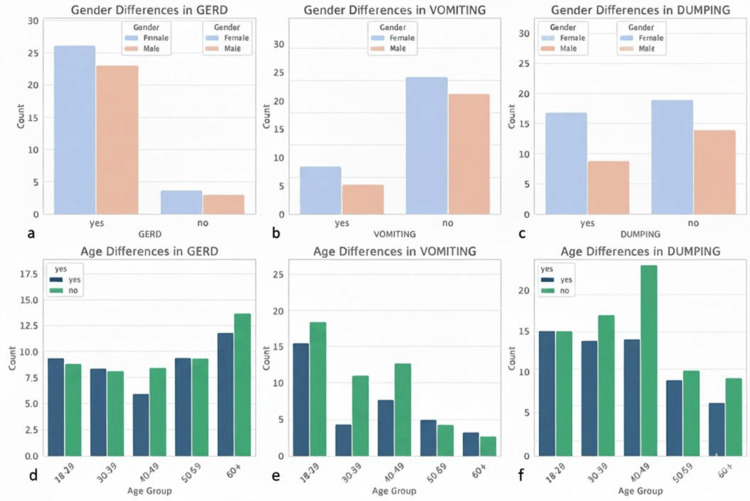
Frequency of postoperative complications (GERD, vomiting, and dumping syndrome) by patient gender and age group The top panels (a-c) compare the incidence of these conditions between female and male patients. Concurrently, the bottom panels (d-f) display the age-based distribution across five groups, distinguishing between patients. Data are expressed as count (n) GERD: gastroesophageal reflux disease; LSG: laparoscopic sleeve gastrectomy

The analysis of sex and age differences in symptoms among patients who successfully completed Ramadan fasting after bariatric surgery revealed notable trends. The sex-based comparison indicates that the prevalence of GERD, vomiting, and dumping syndrome varies between males and females, with some symptoms appearing more frequently in one sex. This suggests potential physiological or behavioral differences in postsurgical responses to fasting. Additionally, the age-based distribution highlights the fact that symptoms are not uniformly experienced across different age groups. Older patients, particularly those above 40 years, exhibit a higher frequency of symptoms compared to younger individuals, which may be attributed to reduced physiological adaptation or pre-existing health conditions. The findings emphasize the importance of individualized medical guidance for post-bariatric patients observing fasting, considering both sex and age-related variations in symptom occurrence (Figure 5).

**Figure 6:**
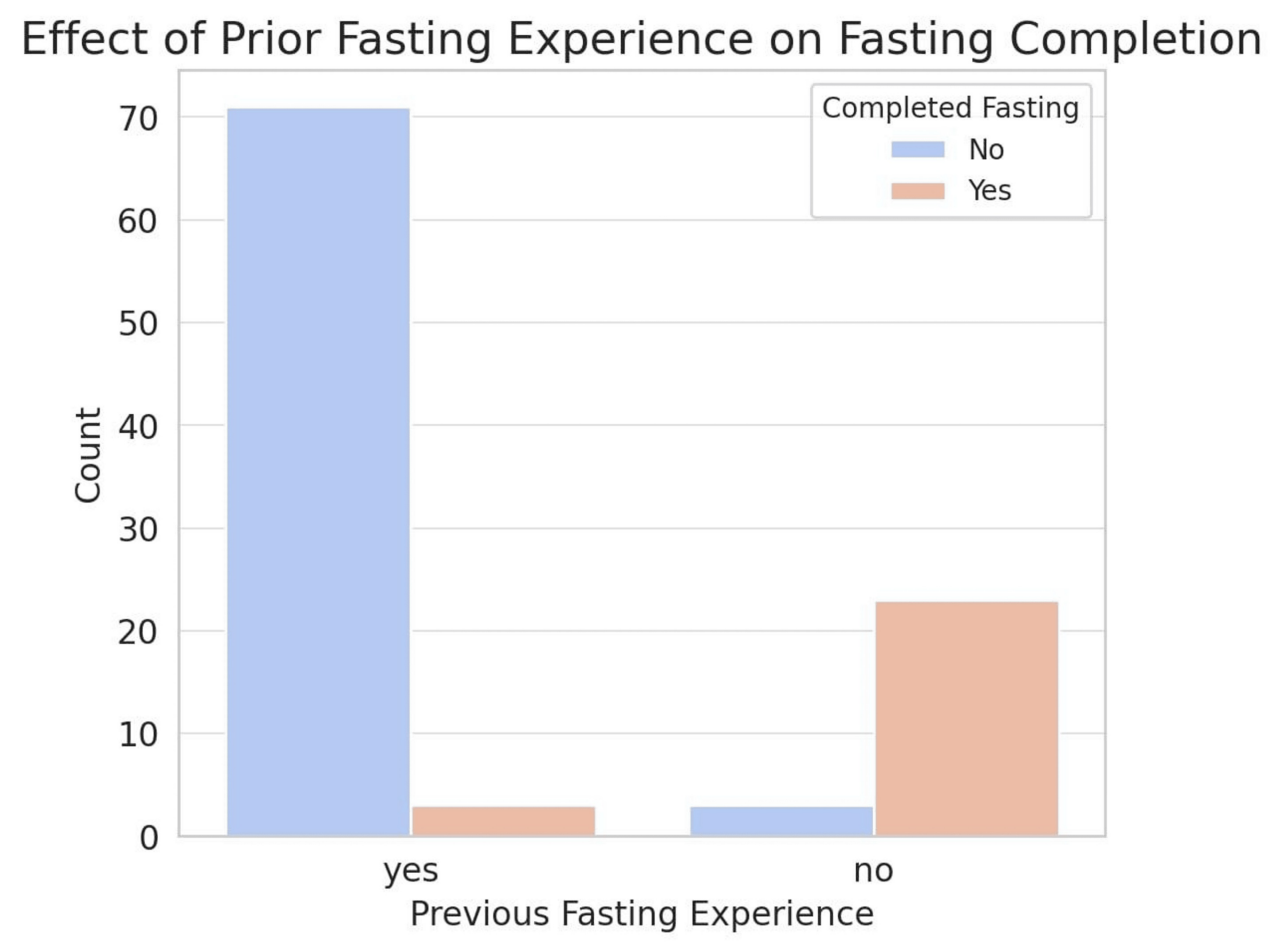
Effect of prior fasting experience on Ramadan fasting completion among patients who underwent LSG Data are presented as the count (n) of patients who successfully completed fasting LSG: laparoscopic sleeve gastrectomy

Analysis of prior fasting experience and its impact on fasting completion among post-bariatric surgery patients revealed a notable relationship. The study results demonstrate that individuals with previous fasting experience were more likely to successfully complete the full month of Ramadan fasting than those without such experience (Figure 6). This suggests that familiarity with fasting, including its physiological and behavioral adaptations, may play a crucial role in postsurgical fasting success. Patients who had not fasted before bariatric surgery exhibited a higher rate of incomplete fasting, possibly due to a lack of prior metabolic conditioning or difficulties in managing dietary intake during prolonged fasting hours. These findings emphasize the importance of pre-fasting counseling and gradual adaptation strategies for post-bariatric patients attempting Ramadan fasting, especially those without previous fasting experience.

## Discussion

This study investigated the effects of Ramadan fasting in patients who underwent LSG. The study findings indicate that the ability to fast successfully during Ramadan is significantly influenced by the time elapsed since the surgery. Patients in the three-month post-surgery group faced challenges in completing the fast, whereas those in the 12-month post-surgery group reported higher success rates. This observation aligns with recommendations suggesting that patients should avoid fasting during the first 6-12 months after surgery to prevent malnutrition and medical complications [8-10]

A notable concern in our study was the high prevalence of GERD, which affected patients in the ≥12 months group. This finding is consistent with reports indicating that upper gastrointestinal symptoms, including GERD, are common during fasting after metabolic and bariatric surgery [11,12]. However, some studies suggest that Ramadan fasting may improve GERD symptoms, although further research is needed to validate these results. Bohamad et al. [13] assessed the severity of GERD symptoms during Ramadan and found that fasting may lead to symptom improvement. Mardhiyah et al. [14] indicated that GERD symptoms were less severe during Ramadan compared to non-fasting periods. Their study concluded that fasting during Ramadan might alleviate the symptoms of GERD.

In addition to GERD, symptoms such as vomiting and dumping syndrome were also observed in our cohort. Dumping syndrome, which is characterized by rapid gastric emptying, is a known complication of bariatric surgery [15]. An encouraging finding was the absence of vitamin and mineral deficiencies among our study participants despite a prolonged fasting period. This contrasts with the concerns that fasting after bariatric surgery can lead to malnutrition [8]. The current study results suggest that with proper guidance, patients can maintain an adequate nutritional status during Ramadan fasting.

In addition, the question of when Ramadan will occur should be proactively addressed by the upper GI and bariatric multidisciplinary team (MDT) to ensure timely planning and effective counseling for patients who intend to fast during Ramadan while undergoing bariatric surgery. Further research is necessary to evaluate the impact of various bariatric procedures, particularly Roux-en-Y gastric bypass (RYGB), on individuals who fast, as these surgeries may influence nutritional status during periods of fasting.

## Conclusions

This retrospective observational study evaluated the optimal timing of fasting after LSG to guide patients to safely observe Ramadan. The study analyzed the experiences of 100 patients who underwent LSG at varying durations before Ramadan in Egypt. The findings revealed that fasting completion rates were significantly influenced by the time elapsed since surgery, with patients who underwent LSG 12 months or more before Ramadan having the highest success rates. GERD prevalence increased with time after surgery, particularly in the ≥12-month group, whereas vomiting and dumping syndromes exhibited a more stable pattern over time. No instances of calcium, vitamin D, or vitamin B deficiency were observed, and dehydration did not lead to hospitalization. Sex and age differences related to the prevalence of symptoms were noted, with older patients exhibiting a higher frequency of symptoms. Prior fasting experiences positively impacted fasting completion rates. These findings have clinical implications for preoperative counseling and postoperative dietary guidance, particularly for patients seeking to safely observe Ramadan fasting after LSG. This study highlights the importance of adequate postoperative recovery, long-term symptom monitoring, and individualized medical guidance for post-bariatric patients during Ramadan fasting.
